# Different Delivery Modalities of Virtual Reality Training in Emergency Medicine: Systematic Review

**DOI:** 10.2196/84310

**Published:** 2026-07-31

**Authors:** Marianna Costa, Martina Codato, Giorgia Brugnera, Silvia Galiazzo, Elisabetta Cantele, Todd P Chang, Silvia Bressan

**Affiliations:** 1 Department of Women’s and Children’s Health, University of Padua, Via Giustiniani 3, Padova, 35128, Italy, 39 049 8213570; 2 Children’s Hospital Los Angeles, University of Southern California, Los Angeles, CA, United States

**Keywords:** simulation, virtual reality, VR delivery modalities, emergency training, educational strategies

## Abstract

**Background:**

Simulation is a cornerstone of emergency medicine education, and virtual reality (VR) has emerged as a promising immersive and scalable training modality. However, it remains unclear which VR instructional design features most effectively support learning outcomes.

**Objective:**

We aimed to provide a systematic overview of the literature on immersive VR delivery modalities in prehospital and in-hospital emergency medicine training, focusing on how different instructional design features influence educational effectiveness.

**Methods:**

We conducted a systematic review according to the PRISMA (Preferred Reporting Items for Systematic Reviews and Meta-Analyses) 2020 guidelines, including English-language articles examining the use of VR for the training of health care professionals, specifically randomized controlled trials (RCTs), non-RCTs, and prospective or retrospective observational comparative studies. PubMed/MEDLINE, Scopus, Embase, and CINAHL were searched up to January 28, 2026. We included studies evaluating immersive headset-based VR for emergency medicine training among qualified health care professionals. Studies involving students, laypersons, nonemergency settings, or augmented, mixed, or web-based reality were excluded. Risk of bias was assessed using the Joanna Briggs Institute critical appraisal tools. Given the wide heterogeneity in methodology across studies, a meta-analysis was not performed, and findings were synthesized using a structured narrative approach based on VR delivery modalities.

**Results:**

Six comparative studies in emergency medicine training were included from 1770 identified records. The included studies compared different immersive VR delivery modalities, including varying exposure schedules, software with or without gamification, machine-guided versus educator-guided training, active versus observational participation with different persuasive feedback strategies, and VR with or without manikin integration. Educational outcomes were heterogeneous, including knowledge retention, procedural performance, and learner-related measures. Overall, the included studies were of moderate to high methodological quality. While observational studies were more susceptible to selection bias, RCTs demonstrated adequate randomization and statistical analysis. Across the included studies, repeated and temporally distributed VR exposure consistently improved knowledge retention and performance, whereas gamification, increased persuasive feedback, educator-guided delivery, and manikin integration showed inconsistent or no additional educational benefit.

**Conclusions:**

Unlike previous reviews focusing on the overall educational value of VR, this review compared different immersive VR delivery strategies in emergency medicine training. The few studies identified suggest that structured and repeated engagement with VR simulations may enhance learning effectiveness, whereas additional technological features do not consistently improve outcomes. The available evidence is limited by the small number of studies, heterogeneous interventions and outcome measures, and moderate risk of bias, restricting the strength of the conclusions. These findings suggest that optimizing instructional design may be more important than increasing technological complexity when developing immersive VR educational programs. Further high-quality studies with standardized outcome measures are needed to identify the most effective VR training strategies for emergency medicine education.

## Introduction

Emergency care represents a particularly high-risk setting, characterized by operational complexity, intense time pressure, rapidly evolving situations, and dynamic team structures [1]. For this reason, it is important that both health care professionals working in this field and personnel in training receive proper and repeated emergency training. In some institutions, the number of residents has increased over the years, leading to varying levels of preparedness for critical events and reduced opportunities for direct emergency clinical experience [2-4]. As a result, many residents receive less exposure to emergency scenarios than required, raising concerns that they may not acquire the necessary competencies during their training.

Limited exposure to time-critical emergencies has highlighted the need to expand simulation-based medical education. Simulation has become an essential component of emergency medicine education, providing a safe, controlled environment that ensures psychological safety and enhances skill retention. This educational approach enables health care professionals to practice managing high-stakes scenarios at various levels of complexity, improving their readiness to respond effectively in real-life emergencies [5].

Different simulation techniques and delivery modalities have been employed for various training purposes; as a matter of fact, one educational approach cannot adequately cover trainees’ needs at each learning level. Substantial empirical research concerning conventional manikin-based simulation underscores the effectiveness of simulation training compared to traditional educational methods [5]. Manikin-based simulation is recognized as a useful and safe technique; the modalities to conduct health care simulation scenarios (location, equipment, and human resources needed) according to the planned learning objectives are well described [6]. Although manikin simulation has advantages (skill practice, multidisciplinary team training), it requires significant resources (instructors, medical equipment, and a dedicated setting) to be carried out [6].

Virtual reality (VR) represents a more recent technology for simulation training in medical education [7-9]. It is defined as an immersive technology that reproduces an environment that completely replaces the physical context with a 3-dimensional digital space, allowing the user to engage in a simulated setting for a temporary experience, often within a safe or controlled area. Through its hardware components, such as head-mounted displays and joysticks, and software, VR enables trainees to immerse themselves in scenarios that closely match real-life settings and to tackle progressively challenging tasks through tailored learning paths [10].

Since virtual simulation can be self-administered, trainees can access virtual clinical or surgical settings anywhere at any time and develop self-reliance in specialized areas, such as pediatric emergency care. There has been a significant expansion of interest in research on VR and its many applications in medical education. The effects of VR-based training on health care professionals have mostly been studied in relation to the improvement of technical skills, especially in the surgical field (laparoscopic surgery or robot-assisted surgery, and procedural practice) [11,12].

In recent years, simulation through VR in health care has expanded as an educational tool also for nontechnical skills training (eg, decision making, leadership, and situation awareness) [12]. A recent review comparing VR training with traditional manikin-based simulation for health care professionals found that VR experiences are most effective when proctored [13]. Specifically, Foronda et al [13], drawing on both the literature and experience, recommend providing learners with guidance prior to VR simulations—such as instruction on equipment use, preparatory briefings, opportunities for questions, and facilitated debriefing—to enhance the effectiveness of new technology–based training.

Although the use of VR in medical emergency simulation is expanding, little is known about the effectiveness of different delivery modalities of VR simulation. The question is not *if* VR is effective, but *how* VR can be most effective. This is an important area of investigation to identify the optimal balance between educational benefit and resource investment. Our systematic review aims to systematically identify and summarize available scientific evidence describing immersive VR delivery modalities used in prehospital and in-hospital emergency medicine training, with the objective of comparing the different approaches currently adopted in VR-based educational interventions in the field of emergency medicine training.

## Methods

### Study Design

This systematic review was conducted in accordance with the PRISMA (Preferred Reporting Items for Systematic Reviews and Meta-Analyses) 2020 guidelines (Checklist 1). The study protocol was registered in the PROSPERO (International Prospective Register of Systematic Reviews; CRD42024507591).

### Search Strategy

Electronic searches were performed in the following databases: PubMed/MEDLINE, Scopus, Embase, and CINAHL (with MEDLINE records excluded within the CINAHL search to minimize duplication across databases) from January 2000 to January 28, 2026. The initial search was conducted on January 4, 2025, and rerun on January 28, 2026, following peer-review feedback, incorporating the suggested modifications to the search strategy.

The search strategy was developed according to the PICO (Population, Intervention, Comparison, and Outcome) framework. The following concepts were combined: population (P)=health care professionals involved in emergency medicine training; intervention (I)=immersive VR-based training; comparison (C)=alternative VR delivery modalities for the same educational purpose; and outcome (O)=effectiveness of VR delivery strategies.

The search strategy was constructed using MeSH, CINAHL Headings, and free-text keywords according to the key research question. No previously published search filters were used, and no search strategies from prior reviews were adapted or reused. It was optimized by an experienced librarian.

The search was limited to English-language publications and to studies published between 2000 and 2025, as contemporary literature was considered most representative of current applications of VR in health care education. Due to indexing lag across databases, records published in 2026 may not yet have been fully indexed at the time of the search update. No study registries were searched. Gray literature sources were not searched, and no additional online or print sources were purposefully browsed. We did not contact corresponding authors, first authors of included studies, or subject-area experts to identify ongoing or unpublished studies. The reference lists of eligible articles were screened to ensure inclusion of all relevant articles. The full search strategies for all databases are reported in Checklist 2 according to the PRISMA-S (Preferred Reporting Items for Systematic Reviews and Meta-Analyses Literature Search Extension) extension for reporting literature searches in systematic reviews. All records identified through database searching were uploaded into the Covidence software for duplicate removal and systematic screening of eligible studies.

### Inclusion Criteria

We included English-language articles focusing on VR used for the training of health care professionals (ie, medical students, residents, clinicians, trainees, assistants, interns, nurses, emergency technicians, paramedics, and prehospital physicians) in prehospital and in-hospital medical emergencies. We examined its application both in the context of formal emergency medicine training programs and, more broadly, in educational interventions designed to enhance the competencies of health care professionals in the management of acute and urgent clinical scenarios. Our review included randomized controlled trials (RCTs), non-RCTs, and observational prospective and retrospective comparative studies.

### Exclusion Criteria

We excluded studies exclusively enrolling laypeople, nonqualified health care workers, or students. Studies focusing on VR training for scenarios different from medical emergencies and studies assessing augmented reality, mixed reality, or web-based platforms instead of head-mounted display VR technology were excluded.

Augmented reality is defined as a technology that extends or enhances the real physical environment with superimposed virtual content, enabling hybrid experiences that combine real and digital elements, accessible in everyday contexts through mobile devices, wearables, or projectors, whereas mixed reality has been defined as any user experience combining real and virtual objects, with mixed reality’s 2 subforms being augmented reality and augmented virtuality [10].

### Study Selection

Identified records were screened based on the inclusion and exclusion criteria. Two independent reviewers (GB and SG) initially screened the articles based on their titles and abstracts. Eligible papers underwent full-text assessment to identify papers that eventually met the inclusion criteria. Any disagreement during the study selection process was discussed with a third independent reviewer (SB) to resolve discrepancies.

### Data Extraction

Relevant information from the included studies was extracted by 2 independent reviewers (M Codato and EC) using an ad hoc data extraction tool. At this stage, any disagreement on the data to be extracted was resolved through discussion with a third reviewer (SB). We extracted information on the authors, journal, year of publication, country of study conduct, study design, characteristics of the study population, study objectives, VR training characteristics and type of scenarios, VR delivery modalities (intervention/control), and results on the effectiveness of the VR delivery modalities assessed. The outcomes assessed by each included study reflected improvements in both technical skills and nontechnical competencies, such as clinical reasoning, prioritization of interventions, and situational awareness. Effect sizes were interpreted according to Cohen *d* conventional thresholds (small=0.2, moderate=0.5, and large=0.8). Cohen *d* was derived from the reported means and SDs using the standard formula [14].

### Data Analysis

Given the substantial heterogeneity in study designs, interventions, comparators, and outcome measures, a quantitative meta-analysis was not performed. Instead, the findings were synthesized using a structured narrative synthesis informed by the Guidance on the Conduct of Narrative Synthesis in Systematic Reviews [15].

The synthesis proceeded in 3 stages. First, studies were grouped according to the educational domain targeted by the intervention, including technical skills acquisition and recognition of emergency clinical scenarios, cardiopulmonary resuscitation (CPR) training and the approach to clinical emergencies, and collaborative training. Within these domains, studies were further organized according to key VR delivery characteristics, including gamification, frequency and spacing of exposure (distributed vs single-session training), level of immersion and participation (active vs observer-based learning), feedback modality (persuasive vs minimal feedback), and integration with manikin-based simulation or traditional training modalities.

Interpretation of the findings incorporated consideration of methodological quality, risk of bias, and contextual factors, such as participant characteristics, training setting, and variability in outcome measurement.

### Risk of Bias

Risk of bias assessment was performed by 2 independent reviewers. Studies were appraised according to the individual domains of the Joanna Briggs Institute Critical Appraisal Checklists (see Checklist 3). Any disagreements were resolved through the assessment of a third independent reviewer.

The methodological quality of the included studies informed the interpretation of the findings. In the absence of a quantitative synthesis, no formal weighting was applied; however, greater emphasis was placed on findings derived from RCTs with a lower risk of bias. Findings from studies with an unclear or higher risk of bias were interpreted cautiously and considered hypothesis-generating rather than confirmatory.

## Results

### Study Characteristics

Following the initial search, a total of 1770 articles were retrieved, and 52 unique studies were left after duplicate removal and were screened using the Covidence online software.

**Figure 1.**
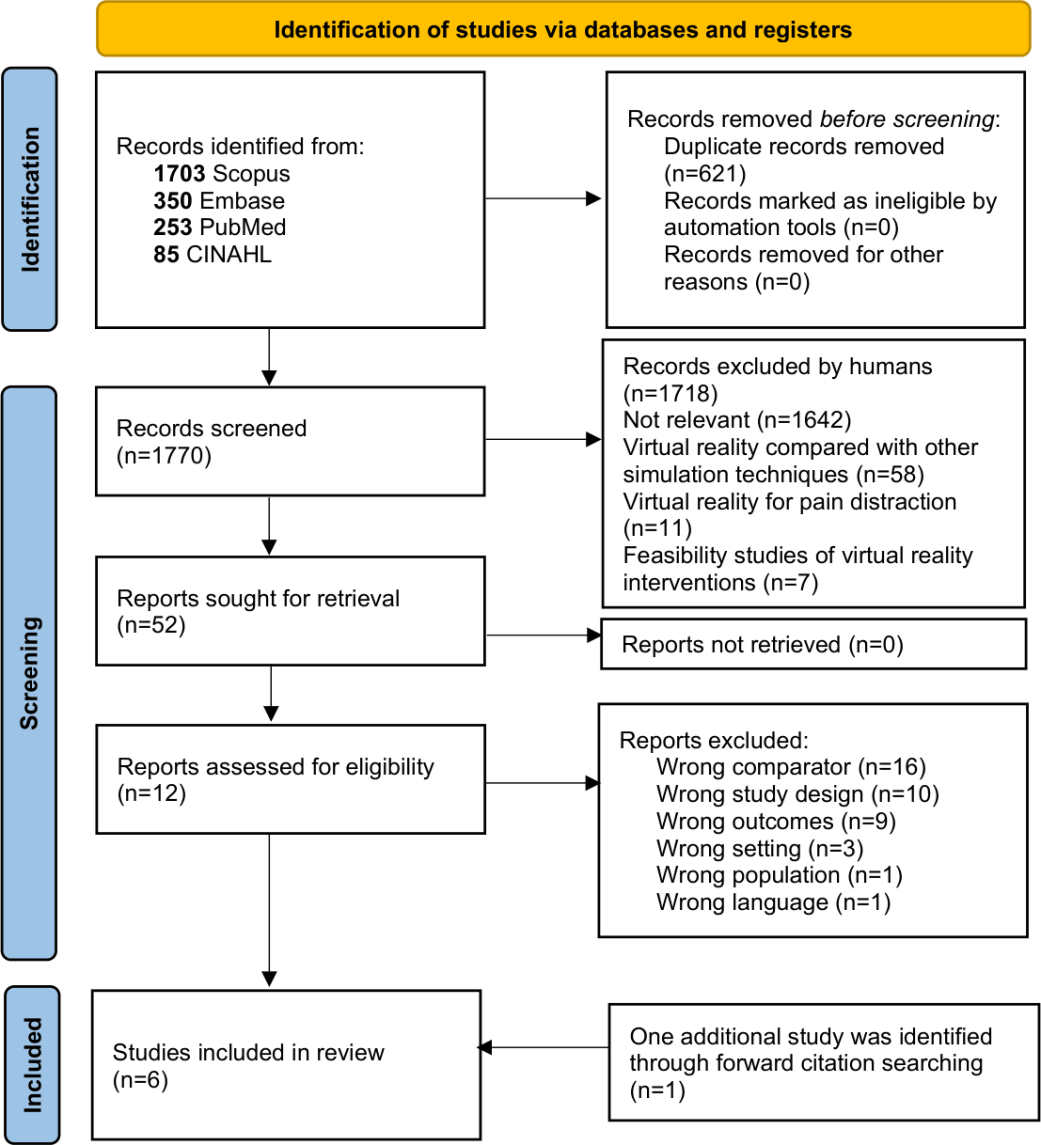
PRISMA (Preferred Reporting Items for Systematic Reviews and Meta-Analyses) 2020 study flow diagram of the study selection process.

The PRISMA 2020 flowchart for the study selection process is shown in Figure 1.

After an independent full-text review based on the inclusion and exclusion criteria, only 5 studies met the inclusion criteria for our systematic review. One additional article was identified through hand-searching of the reference lists of the included articles, increasing the total number of included studies to 6. Their characteristics are described in Table 1. None of the included studies pertained to the prehospital setting.

**Table 1.** Characteristics of included studies investigating virtual reality interventions for technical skills acquisition, cardiopulmonary resuscitation, and the recognition and management of clinical scenarios in emergency medicine.

VR^a^ for technical skills acquisition and systematic recognition/management of clinical emergencies							
Study	Country/study period	Design/setting	Participants	Learning objectives	VR scenario/VR module features	VR delivery modality	
						Intervention	Control
Larsen et al [16], 2023, *Ultrasound in Med & Biol*	Denmark/Oct 2021-Dec 2021	RCT^b^ 2 study arms Double-blinded Allocation ratio 1:1 Single-center	48 students/residents (24 per study arm) No previous experience with FLUS^c^	Train in the use of lung ultrasound (pathology findings detection)	FLUS module (pleural effusion, pneumonia, etc)	Gamified VR module	Nongamified VR module
Lietz et al [17], 2023, *Acta Paed*	Austria/Aug 2021-Mar 2022	Pilot RCT 3 study arms Allocation ratio 1:1:1 Single-center	36 students (12 per study arm)	Improve proficiency in emergency algorithms and tasks; crisis management in high-intensity emergency; CPR^d^ skills	Infant with shock rapidly moving to nonshockable cardiac arrest Single-player	VR training interval after baseline training Group A: monthly for 5 times Group B: once after 3 months Group C: baseline training	No further VR sessions after baseline VR training
Kitapcioglu et al [18], 2024, *JMIR Serious Games*	Turkey/2023‐2024	RCT 2 study arms Single-center	62 students (31 per study arm)	Provide ACLS^e^ training	Both groups undertook VR-based basic training for ACLS. Afterward: MG^f^ group were trained with a VR-based advanced training module EG^g^ group attended VR-based, educator-guided training in the metaverse	VR-EG training after VR basic ACLS training	VR-MG full ACLS training (based + advanced)
Jaskiewicz et al [19], 2020, *Medicine*	Poland/April 2019-May 2019	Cross-over study Single-center	91 medical students completed BLS^h^ course	Assess CPR quality during sudden cardiac arrest scenario (SCA)	3-h BLS course (45 min theoretical background + 90 min of CPR on the manikin) Single-player	2 min hands-on-only CPR VR SCA scenario versus traditional SCA scenario	2 min hands-on-only CPR Traditional SCA scenario versus VR SCA scenario
Mühling et al [20], 2023, *Multimedia System*	Germany/Oct 2020-Jul 2021	Observational study 2 groups (observers and active participants) Single-center	227 medical students 97 active participants, 130 observers (8‐10 students per VR session)	Train cognitive abilities (ie, executive functions, situational awareness, time management, and prioritize tasks)	120-min VR sessions including 3‐4 scenarios and guided by an assistant + debriefing (60 min) of the cases with a tutor (ie, sepsis, acute myocardial infarction, etc) Single-player	Active participants Only 1 student per scenario (3‐4 students per VR session) was exposed to the VR environment	Observers watched from the first-person perspective on a monitor and were able to assist verbally the colleague student undergoing the VR scenario
Khanal et al [21], 2014, *J Biomed Informatics*	United States/—	RCT 3 study arms Single-center	156 ACLS-certified clinicians (26 teams of 6 participants)	Provide team ACLS training	VF^i^ or PEA^j^ case scenarios (5 min ACLS team training + 30 min) VR groups received training in a VR environment (20 min guided single-user tutorial to familiarize with the user interface) Multiplayer	Persuasive VR ACLS training (feedback components available for all team members, besides alerts specific to individual member roles) Minimally persuasive VR ACLS training (limited feedback components)	Traditional manikin-based in-person training

a VR: virtual reality.

b RCT: randomized controlled trial.

c FLUS: focus lung ultrasound.

d CPR: cardiopulmonary resuscitation.

e ACLS: advanced cardiac life support.

f MG: machine guidance.

g EG: educator guidance.

h BLS: basic life support.

i VF: ventricular fibrillation.

j PEA: pulseless electrical activity.

As indicated in Table 1, medical students were the primary study population in 4 of the 6 included studies. The VR scenarios, the outcomes, and the delivery modalities assessed were different.

Three of the included RCTs investigated the use of VR for technical skills acquisition, disease recognition, and management of clinical emergencies, operationalizing the intervention through distinct modalities. These included varying time intervals between VR training sessions [15], the use of VR software with or without gamification elements [17], and educator-guided versus machine-guided VR sessions [16].

Two studies implemented the use of VR for CPR training [18] and for the approach to clinical emergencies, comparing the use of VR with or without a manikin during CPR and passive versus active exposure to VR-based training, respectively [19]. Only 1 study described collaborative VR sessions delivered through VR software incorporating varying levels of persuasive feedback intensity.

As shown in Table 2, the learning outcomes and the measures of effectiveness assessed by the included studies were also diverse.

**Table 2.** Outcomes, measurement instruments, and effect sizes of included studies evaluating virtual reality interventions in technical skills training, cardiopulmonary resuscitation performance, and clinical scenario management in emergency medicine.

Study	Country	Outcomes	Measures of outcome	Key findings	Cohen *d* for learning outcomes
Virtual reality for technical skills acquisition and systematic recognition and management of clinical emergencies					
Larsen et al [16], 2022, *Ultrasound in Med & Biol*	Denmark	Primary*:* Educational impact of novice FLUS^a^ users participating in a gamified/nongamified VR^b^ training module in FLUS Secondary: Assessment of learning effect by comparing the scores of each group with known test scores of novices, intermediates, and experienced users in FLUS	Assessment by simulation test on US Simulator Test scores were calculated as the sum of points, given for correct assessment of final diagnosis in each case. A correct final diagnosis=2 points, partially correct=1 point, and incorrect=0 points	No difference was found between gamified (mean=15.5 points, 95% CI 14.5‐16.5) and nongamified (mean=15.2 points, 95% CI 14.3‐16.1) Gamified elements did not affect learning outcome (mean, SD): gamified: 15.5 (SD 2.4) vs nongamified: 15.2 (SD 2.1*; P*=.66) Known test score on US mentor simulator lung ultrasound (mean, SD) Novices: 12 (SD 3.46); intermediate: 15.7 (SD 1.74); experienced: 17 (SD 0.85) Gaming/nongaming learning effect: most comparable test score for known competence groups in lung ultrasound Novice: Both VR groups had significantly higher test scores than known novice (gamified: *P*<.001 vs nongamified: *P*<.001) Intermediate: Both VR groups had test scores that were not signiﬁcantly different from those of known intermediates (gamified: *P*=.63 vs nongamified: *P*=.24) Expert: Both VR groups had signiﬁcantly lower test scores than known experts (gamified: *P*<.001 vs nongamified: *P*<.001)	<0.2 (small)
Lietz et al [17], 2023, *Acta Paed*	Austria	Primary*:* Individual performances after each VR session Secondary: Evaluation of subjective stress and workload after each training session User experience	Individual performance score (15 variables coded into the program and including all measures sets and the duration and initial timing of each intervention) NASA Task Load score Questionnaire regarding various aspects of participants’ individual experiences with the VR headsets after each training session	Group A had the highest score improvement compared with the other 2 study groups Assessment training (mean, SD): Group A^c^: 11.08 (SD 1.84) vs Group B^d^: 8.54 (SD 1.85*; P*=.004, Cohen *d*=1.38) Group A: 11.08 (SD 1.84) vs Group C^e^: 9.25 (SD 1.80*; P*=.03) The average perceived mental demand of participants in Group A after the baseline training was 11.92 (SD 4.92) out of 20 points (*P*=.42). It reduced significantly in the final assessment training to 8.17 (SD 4.59*; P*=.06) Compared satisfaction with individual performance in Group A: improvement of 0.67 from a mean score of 2.17 (SD 0.83) to 1.50 (SD 0.90); Group A felt the most comfortable during the VR simulation, with an improvement of 0.92 from a mean score of 2.75 (SD 1.48) to 1.83 (SD 0.83) Participants stated that VR interface was easy to use (mean, SD): Group A (1.25, SD 0.83), Group B (1.50, SD 0.67), Group C (1.75, SD 0.62)	1.38 (vs one-time VR) 1.01 (vs control group) (large)
Kitapcioglu et al [18], 2024, *JMIR Serious Games*	Turkey	Primary: Comparison of VR ACLS^f^ test scores between the MG^g^ and EG^h^ groups	Virtual reality examination score Duration of MG vs EG training for each participant Correlation between examination scores and time spent on training	Virtual reality exam score (total 100 points): MG group (mean, SD) (53, SD 33) vs EG group (mean, SD) (68, SD 35*; P*=.08) Time spent for MG vs EG training (in min): MG group (median, range) (66, range 13-100) vs EG group (median, range) (15, range 5-30; *P*=.002) MG group rho=0.569, *P*=.005 vs EG group rho=0.298 and *P*=.10. The post hoc power analysis (80%) supported these findings	0.44 (moderate)
Virtual reality for CPR training and approach to clinical emergencies					
Jaskiewicz et al [19], 2020, *Medicine*	Poland	Primary*:* Assess CCs^i^ quality during a SCA^j^ scenario performed with the use of VR prototype Secondary*:* Assess if additional equipment may cause CCs subjectively harder to provide and analyze impressions of students	CCs were conducted on CPR mannequin; quality was measured with SimPad equipped with Skillreporter software (Laerdal, Stavanger, Norway) Five questions regarding previous VR experience Questionnaire about the applicability of VR in the resuscitation training	No statistically significant differences between the VRS^k^ and TS^l^ groups neither in CCs rate (mean, SD): TS 114.9 (SD 11.8) vs VRS 114.2 (SD 11.9) nor in CCs depth (mean, SD): TS 49.3 (SD 7.4) vs VRS 47.8 (SD 8.8). A statistically significant difference was found in the percentage of FCR^m^ component (for VRS group mean 60.2, SD 37.5 vs TS group mean 83.7, SD 27.9; *P*<.001). Majority of participants (97/99, 97.8%, 95% CI 0.92‐0.99; *P*<.01) rated the educational value of VR in CPR training at 3 or 4 points in 1- to 4-point scale (1=very low, 4=very high)	0.71 (moderate)
Mühling et al [20], 2023, *Multimedia System*	Germany	Primary*:* Evaluation of STEP-VR^n^ into the medical curriculum Secondary*:* Perception of VR-based training, psychological distress users experience and perceived learning benefits	Questionnaires for evaluation in both groups of: Level of presence Estimated learning success Motivation pretraining and posttraining Questionnaires for evaluation of perceived stress on estimated learning success in AP^o^ group	No differences in demographic characteristics between observers (n=130) and AP (n=97) AP rated the degree of presence significantly higher than observers (mean, SD) (3.81, SD 0.98, n=97 and 3.26, SD 0.74, n=130, respectively, *P*<.001) Estimated learning success was rated higher in AP than observers (mean, SD) (3.87, SD 0.75, n=97 and 3.72, SD 0.77, n=130, respectively, *P*=.05) Motivation pretraining was not significant, posttraining (mean, SD) 3.61, SD 0.84 and 3.07, SD 0.88, *P*>.01 No statistically significant correlations were identified	0.20 (small)
Collaborative VR training					
Khanal et al [21], 2014, *J Biomed Informatics*	United States	Primary*:* Team performance tested during high-fidelity simulation performed in front of ACLS human experts Pretest phase**:** assessment of baseline performance (directly observing teams) Posttest phase**:** assessment of performance after the intervention (directly observing teams and reviewing video-recorded scenario)	Pre/postintervention tests Pretest phase**:** time to perform critical actions Posttest phase**:** electronic checklist assessment tool developed and validated by a team of expert ACLS trainers. It included items that correspond to AHA^p^ guidelines for ACLS	Pretest team performance*:* no statistically significant difference between control and persuasive groups. AHA guidelines adherence (141/360, 39.4%); of total 360 tasks: control: 39.1% (47/120), persuasive: 35.8% (43/120), minimally persuasive: 43.3% (52/120) Posttest team performance*:* increased AHA guidelines adherence (58.3%); of total 360 tasks: control: 68.3% (82/120), persuasive 57.5% (69/120), minimally persuasive: 49.1% (59/120) PEA^q^/Vfib^r^/VTach^s^ (control vs minimally persuasive: *P*=.05 and *P*=.02); Pre/post comparison of performances of groups showed significantly improved performances for control (*P*=.02 for PEA and *P*=.01 for VFib/VTach) and persuasive groups (*P*=.02 for PEA, *P*=.048 for VFib/VTach)	N/A^t^

a FLUS: focus lung ultrasound.

b VR: virtual reality.

c Group A: monthly training for 5 months.

d Group B: once after 3 months.

e Group C: baseline training.

f ACLS: advanced cardiac life support.

g MG: machine-guided.

h EG: educator guided.

i CCs: chest compressions.

j SCA: sudden cardiac arrest.

k VRS: virtual reality scenario.

l TS: traditional scenario.

m FCR: full chest relaxation.

n STEP-VR: Simulation-Based Training of Medical Emergencies for Physicians.

o AP: active participants.

p AHA: American Heart Association.

q PEA: pulseless electrical activity.

r VFib: ventricular fibrillation.

s VTach: ventricular tachycardia.

t N/A: not applicable.

Regarding the use of VR for technical skills acquisition, disease recognition, and the management of clinical emergencies, no significant differences were identified in the RCTs comparing gamification strategies [17] or automated machine-guided versus educator-guided VR training modalities [16].

In contrast, a study examining different temporal distributions of VR training exposure demonstrated significant differences in outcomes [15]. Specifically, monthly VR training sessions conducted over 5 months resulted in superior individual performance scores compared with 2 sessions delivered within a 3-month period [15].

With respect to CPR training, the study findings indicated no statistically significant differences between groups in terms of chest compression rate or depth. However, a statistically significant difference was observed in full chest recoil, favoring the control group that initially underwent traditional CPR simulation training [18].

Similarly, considering the approach to emergencies as a learning objective, comparisons between active and passive VR exposure revealed that passive participation—defined as observing the VR scenario from a first-person perspective via a monitor while peers engaged in the VR training—was associated with lower self-reported learning success, as measured by an ad hoc questionnaire [19].

Finally, the study investigating varying intensities of persuasive feedback embedded within VR software during collaborative VR sessions found no significant differences between the study groups [20]. To further synthesize findings across studies, results were organized according to VR delivery modalities, focusing on shared instructional design characteristics rather than individual study outcomes (Table 3).

**Table 3.** Synthesis of virtual reality modalities.

Virtual reality delivery modality	Study	Effect on learning	Interpretation
Gamification	Larsen et al [16]	No consistent effect	Not determinant
Distributed exposure	Lietz et al [17]	Positive	Likely beneficial
Active participation	Mühling et al [20]	Positive	Enhances engagement
Feedback intensity	Khanal et al [21]	Mixed	Context-dependent
Educator involvement	Kitapcioglu et al [18]	No clear benefit	Not decisive
Manikin integration	Jaskiewicz et al [19]	Neutral	No added value

### Quality Assessment

The summary risk-of-bias assessment of the included studies is displayed in Table 4.

Four of the 6 included studies had an unclear risk of bias in one or more domains. The evaluation of bias related to participant retention (question 10) was not applicable to most included studies. Only one RCT [17] and one observational study [19] had a low risk of bias. Findings from studies with a higher risk of bias must be interpreted with caution.

**Table 4.** Risk of bias assessment of included studies evaluating virtual reality–based educational interventions.

Study	1	2	3	4	5	6	7	8	9	10	11	12	13^a^
RCT^b^													
Khanal et al [21], 2014	?^c^	?	+^d^	+	−^e^	+	−	+	+	N/A^f^	+	+	+
Larsen et al [16], 2023	+	+	+	+	+	+	+	+	+	N/A	+	+	+
Lietz et al [17], 2023	+	+	+	?	?	+	?	+	+	+	+	+	+
Kitapcioglu et al [18], 2024	+	−	?	−	+	?	+	+	+	N/A	+	+	+
Observational study													
Jaskiewicz et al [19], 2020	+	+	+	−	−	?	+	N/A	N/A	N/A	+	N/A	N/A
Mühling et al [20], 2023	+	+	+	+	+	+	+	N/A	N/A	N/A	+	N/A	N/A

a Numbered column headings (1-13) represent the corresponding questions (Items 1-13) of the Joanna Briggs Institute Critical Appraisal Checklist used to assess the methodological quality of the included studies.

b RCT: randomized controlled trial.

c ?: Unclear.

d +: Yes (criterion met).

e –: No (criterion not met).

f N/A: not applicable.

## Discussion

This systematic review aimed to compare different VR delivery modalities for emergency medicine training, focusing on how specific instructional design features influence educational effectiveness. Despite the growing adoption of VR in medical education, only 6 comparative studies met the inclusion criteria, highlighting the limited evidence specifically addressing *how* immersive VR should be delivered rather than whether VR is effective per se. Overall, the available evidence suggests that active learner engagement and repeated, temporally distributed VR exposure are the instructional strategies most consistently associated with improved learning outcomes, whereas additional technological features, including gamification, persuasive feedback, and educator-guided versus machine-guided delivery, did not demonstrate consistent educational benefits. However, the small number of studies, together with their methodological and conceptual heterogeneity, limits the strength of these conclusions and warrants cautious interpretation.

Notably, several excluded studies compared VR with alternative educational approaches (eg, conventional lectures [21], manikin-based simulations [22], video-based training [23], traditional technique guides [24], etc), highlighting how research has focused more on whether VR is effective compared to other teaching techniques than on how VR can best be effective in enhancing learning.

Timely and active VR exposure appeared to be most effective for training. Repetitive VR training exposures over monthly time windows showed a higher dose-response effectiveness in terms of performance compared with longer time windows of 3 months [15]. This observation [15] aligns with broader educational theory and simulation literature. Beyond VR-specific interventions, the importance of training frequency reflects broader educational principles related to spaced learning, which have consistently been associated with improved knowledge consolidation and long-term skill retention in medical education [25,26].

Prior research has consistently demonstrated that repeated exposure to high-fidelity simulation and CPR retraining improves both knowledge and skill retention [27]. While a previous study reported a significant decline in CPR skills within 6 to 12 months after training [28]—suggesting the need for refresher sessions within that timeframe—a more recent scoping review on pediatric CPR retraining strategies indicates that shorter, more frequent refreshers may be more effective for sustaining performance [29].

Although definitive evidence regarding the optimal retraining interval remains limited, even brief monthly refresher sessions, lasting as little as 2 minutes, appear to enhance skill retention and support higher-quality CPR performance over time [29]. While optimal retraining intervals remain unclear, VR appears to offer an effective platform for facilitating distributed learning, enabling repeated, spaced practice to enhance skill acquisition and retention.

The addition of gamification elements did not result in measurable improvements in technical performance, suggesting that engagement-enhancing features alone may not translate into superior learning outcomes [17]. Although gamification did not improve objective technical performance in the included study, previous literature suggests that game elements may still enhance learner engagement, motivation, and training adherence. Their educational effectiveness may therefore depend less on the presence of gamification itself and more on how these elements are pedagogically integrated within the learning experience [30].

Likewise, machine-guided VR training achieved outcomes comparable to educator-guided training [16], raising important considerations regarding scalability and resource allocation.

In settings with limited faculty availability, automated VR modules may represent a feasible alternative without substantial loss of effectiveness. This finding may have important implications for emergency medicine education, where increasing learner volumes, limited faculty time, and restricted access to simulation centers frequently constrain the delivery of simulation-based training. Recent evidence suggests that AI- or machine-guided VR systems may offer scalable and accessible alternatives to traditional facilitator-dependent simulation by supporting autonomous, repeatable, and remotely accessible training experiences without substantial reductions in educational effectiveness [31].

With respect to CPR training, VR alone did not consistently improve objective performance measures, such as compression depth or rate when compared with traditional approaches [18]. These findings suggest that, for psychomotor skills requiring tactile feedback, VR may need to be integrated with physical manikins or complementary training modalities. These findings differ from those of a study that evaluated manual skill acquisition during CPR training using both guided and semiguided VR modes [32]. This paper demonstrated that repeated practice of chest compressions in VR significantly improved performance—particularly compression rate—regardless of whether a manikin was used. However, this study [32] was excluded from the present analysis as it involved undergraduate computer science students, which does not align with the target group of this review.

Conversely, in cognitive domains such as prioritization, situational awareness, and emergency algorithms, VR appears to offer potential benefits, particularly when learners are actively immersed rather than passively observing scenarios [19].

Recent studies have reported VR sessions involving both learners immersed in the virtual environment and others observing the scenario on external displays while contributing in an observational role [33,34].

These approaches may support collaborative learning, reflective observation, and peer-assisted learning, which are recognized educational components of simulation-based training. However, current evidence remains insufficient to determine whether multi-learner immersive formats provide meaningful advantages over simpler single-user models.

Collaborative VR environments and persuasive feedback systems have not consistently demonstrated superior outcomes compared with less complex approaches [20]. This may reflect the still-evolving educational integration of collaborative VR modalities rather than an actual lack of educational benefit.

Notably, few studies [15,19] incorporated structured debriefing into VR-based training, despite its well-established role in simulation-based education. An observational study [19] cites a 60-minute debriefing phase, which occurred after VR-based training; it is described as a tutor-guided review to summarize and discuss problem-solving issues. The articles mentioned above described the debriefing phase as a useful moment for feedback; however, they did not include a comparison between 2 different debriefing modalities.

Debriefing phase in manikin-based simulation has a well-defined role and structure, focusing mainly on nontechnical aspects of clinical case management; in VR simulation, it still seems to have the main aim of providing technical corrections [35,36]. The educational role of debriefing within immersive VR therefore remains poorly defined and represents an important area for future research.

Overall, VR offers important educational advantages, including standardized scenarios, scalability, and flexible access independent of physical simulation centers [37,38]. These characteristics may be particularly relevant in contexts with limited faculty resources or high learner volumes. However, its educational value depends less on technological complexity than on how it is integrated into simulation curricula. Future educational programs should therefore incorporate VR within evidence-based instructional designs that promote active learner engagement, distributed practice, meaningful feedback, and the acquisition of both technical and nontechnical competencies, particularly decision-making in complex emergency scenarios.

This review has several limitations. First, only a small number of studies met the inclusion criteria, limiting the robustness of comparisons across modalities. Second, substantial conceptual and methodological heterogeneity was present in interventions, comparators, and outcome measures, preventing quantitative synthesis. Additionally, a risk of bias was present in several studies, particularly in blinding and allocation procedures. Third, most studies were conducted among undergraduate medical students, limiting generalizability to practicing clinicians and multidisciplinary emergency teams. Fourth, many outcomes were subjective or based on short-term assessments, and none evaluated long-term retention or patient-level clinical outcomes. Lastly, only a few studies explored the cost-effectiveness of various VR modalities. These limitations should be considered when interpreting the present findings and highlight the need for more standardized comparative research.

Unlike previous reviews focusing on the overall educational value of VR, this review compared different immersive VR delivery strategies in emergency medicine training.

By systematically differentiating and comparing distinct VR delivery modalities, this review addresses a critical gap in the literature. Although interest in VR in this field has grown substantially, robust comparative evidence across different delivery modalities remains scarce and fragmented. The few studies identified suggest that timely and structured engagement with VR-based training may enhance educational outcomes. Collectively, this work contributes to advancing simulation-based education and supports the development of more effective, scalable, and strategically implemented emergency training programs. Further high-quality research is needed to determine the most effective VR strategies for emergency medicine education. Rather than investing primarily in increasingly sophisticated VR technologies, future educational programs should prioritize evidence-based instructional design principles that promote active participation, distributed practice, and meaningful feedback. Standardized outcome measures and multicenter comparative trials will be essential to establish best practices for immersive VR implementation in emergency medicine education.

## Supplementary material

10.2196/84310 Checklist 1 PRISMA checklist.

10.2196/84310 Checklist 2 PRISMA-S checklist.

10.2196/84310 Checklist 3 Joanna Briggs Institute checklist.

## Abbreviations

CPR: cardiopulmonary resuscitation
PICO: Population, Intervention, Comparison, and Outcome
PRISMA: Preferred Reporting Items for Systematic Reviews and Meta-Analyses
PRISMA-S: Preferred Reporting Items for Systematic Reviews and Meta-Analyses Literature Search Extension
PROSPERO: International Prospective Register of Systematic Reviews
RCT: randomized controlled trial
VR: virtual reality
