# Supplementary material for: Different Delivery Modalities of Virtual Reality Training in Emergency Medicine: Systematic Review
Source: JMIR XR Spat Comput. 2026 Jul 31;3:e84310. doi: 10.2196/84310 (PMC13427254; doi:10.2196/84310)
Supplement: Checklist 2 [file xr-v3-e84310-s002.pdf]

PRISMA-S Checklist: Reporting of Literature Search Methods

**INFORMATION SOURCES AND METHODS**		
Database name	1	The following electronic databases were searched: MEDLINE (Ovid), CINAHL (EBSCOhost), EMBASE (Ovid), PubMed (MEDLINE interface), Scopus (Elsevier)
Multi-database searching	2	Electronic searches were conducted in CINAHL, explicitly excluding any records already retrieved from MEDLINE to ensure non-duplication across databases
Study registries	3	No study registries were searched.
Online resources and browsing	4	No additional online or print sources were purposefully searched or browsed.
Citation searching	5	Reference lists of included articles were manually screened to identify additional studies.
Contacts	6	We did not contact corresponding or first authors of included trials, or subject-area experts for information about ongoing studies.
Other methods	7	No supplementary search methods were applied.
**SEARCH STRATEGIES**		
Full search strategies	8	*PubMed/Medline*<br><br>(student*[Title/Abstract] OR "Students"[MeSH] OR resident*[Title/Abstract] OR "resident" OR "Internship and Residency"[MeSH] OR clinician*[Title/Abstract] OR physician*[Title/Abstract] OR "Physicians"[MeSH] OR trainee*[Title/Abstract] OR nurse*[Title/Abstract] OR paramedic* [Title/Abstract] OR "paramedic*"[MeSH] OR "Emergency Technician*"[Title/Abstract] OR "Emergency Technician"[MeSH] OR "pre-hospital physician*" OR "pre-hospital physician*"[MeSH] OR "Nurses" [MeSH] OR intern*[Title/Abstract]) AND (emergenc*[Title/Abstract] OR "Emergency Medicine"[MeSH] OR "Cardiac Arrest"[MeSH] OR cardiac arrest[Title/Abstract] OR "heart arrest"[Title/Abstract] OR resuscitation [Title/Abstract] OR "Resuscitation"[MeSH]) AND ("Virtual Reality"[MeSH] OR "virtual reality"[Title/Abstract] OR "virtual simulation"[Title/Abstract] OR "digital simulation"[Title/Abstract]<br><br>OR "extended reality"[Title/Abstract])<br><br>*CINHAL*<br><br>((TI student* OR AB student*) OR (MH "Students") OR (TI resident* OR AB resident*) OR "resident" OR (MH "Internship and Residency") OR (TI clinician* OR AB clinician*) OR (TI physician* OR AB physician*) OR (MH "Physicians") OR (TI trainee* OR AB trainee*) OR (TI nurse* OR AB nurse*) OR (TI paramedic* OR AB paramedic*) OR (MH "paramedic*") OR (TI

		"Emergency Technician*" OR AB "Emergency Technician*") OR (MH "Emergency Technician") OR "pre-hospital physician*" OR (MH "pre-hospital physician*") OR (MH "Nurses") OR (TI intern* OR AB intern*)) AND ((TI emergenc* OR AB emergenc*) OR (MH "Emergency Medicine") OR (MH "Cardiac Arrest") OR (TI cardiac arrest OR AB cardiac arrest) OR (TI "heart arrest" OR AB "heart arrest") OR (TI resuscitation OR AB resuscitation) OR (MH "Resuscitation")) AND ((MH "Virtual Reality") OR (TI "virtual reality" OR AB "virtual reality") OR (TI "virtual simulation" OR AB "virtual simulation") OR (TI "digital simulation" OR AB "digital simulation") OR (TI "extended reality" OR AB "extended reality"))<br><br>AND (PY 2000-2025) AND (LA English)<br><br>*Scopus*<br><br>(TITLE-ABS-KEY (student*) OR TITLE-ABS-KEY (resident*) OR TITLE-ABS-KEY (resident) OR TITLE-ABS-KEY (clinician*) OR TITLE-ABS-KEY (physician*) OR TITLE-ABS-KEY (trainee*) OR TITLE-ABS-KEY (nurse*) OR TITLE-ABS-KEY (paramedic*) OR TITLE-ABS-KEY ("Emergency Technician*") OR TITLE-ABS-KEY ("pre-hospital physician*") OR TITLE-ABS-KEY (intern*)) AND (TITLE-ABS-KEY (emergenc*) OR TITLE-ABS-KEY ("emergency medicine") OR TITLE-ABS-KEY ("cardiac arrest") OR TITLE-ABS-KEY ("heart arrest") OR TITLE-ABS-KEY (resuscitation)) AND (TITLE-ABS-KEY ("virtual reality") OR TITLE-ABS-KEY ("virtual simulation") OR TITLE-ABS-KEY ("digital simulation") OR TITLE-ABS-KEY ("extended reality"))<br><br>AND PUBYEAR > 1999 AND PUBYEAR < 2026 AND (LIMIT-TO (LANGUAGE, "English"))<br><br>*Embase*<br><br>((student* or resident* or resident or clinician* or physician* or trainee* or nurse* or paramedic* or "Emergency Technician*" or "pre-hospital physician*" or intern*) and (emergenc* or "emergency medicine" or "cardiac arrest" or "heart arrest" or resuscitation) and ("virtual reality" or "virtual simulation" or "digital simulation" or "extended reality")).ti,ab,kw.<br><br>limit 1 to english language and "remove medline records" and yr="2000 - 2025") yr="2000- 2025"
Limits and restrictions	9	The search was limited to English-language publications and to studies published between 2000 and 2025, as contemporary literature is considered most representative of current applications of virtual reality in education.
Search filters	10	The search strategy did not incorporate any previously published search filters
Prior work	11	No search strategies from other literature reviews were adapted or reused.
Updates	12	The search was rerun following peer-reviewer feedback, incorporating suggested modifications to the search string.

Dates of searches	13	A comprehensive literature search was initially run on 4 January 2025 and then rerun on 28 January 2026
**PEER REVIEW**		
Peer review	14	The search strategies were developed and peer reviewed by a senior expert librarian. The librarian specifically evaluated the structure, syntax, controlled vocabular and overall composition of the search strategy to ensure methodological rigor and comprehensiveness. Following peer-review feedback on the manuscript, the search strategy was further revised and refined in accordance with the reviewers' recommendations before final implementation
**MANAGING RECORDS**		
Total records	15	Systematic searches of four electronic databases yielded 1770 records. The identification, screening, and inclusion process is presented in Figure 1 (PRISMA 2020 flow diagram).
Deduplication	16	All retrieved records were imported into Covidence, and duplicate records were automatically identified and removed before title and abstract screening.
